# Biocompatible Hydrogel Coating on Silicone Rubber with Improved Antifouling and Durable Lubricious Properties

**DOI:** 10.3390/gels10100647

**Published:** 2024-10-11

**Authors:** Shuai Gao, Zheng Liu, Wei Zeng, Yunfeng Zhang, Fanjun Zhang, Dimeng Wu, Yunbing Wang

**Affiliations:** 1 National Engineering Research Center for Biomaterials, College of Biomedical Engineering, Sichuan University, Chengdu 610065, China; shuai.gao@daxanmed.com (S.G.); zhfanjun@163.com (F.Z.); 2 Chengdu Daxan Innovative Medical Tech. Co., Ltd., Chengdu 611137, China; lz6988@163.com (Z.L.); eden_zengw@163.com (W.Z.); zyf19900320@163.com (Y.Z.)

**Keywords:** silicone rubber, hydrogel coating, antifouling, lubricious

## Abstract

Silicone rubber is widely used in various medical applications. However, silicone rubber is prone to biofouling due to their affinity for lipids and has a high friction coefficient, which can significantly impact their efficacy and performance used as medical devices. Thus, the development of hydrogels with antifouling and lubricious abilities for the modification of silicone rubber is in high demand. We herein prepared a variety of hydrogel coatings mainly based on polyvinylpyrrolidone (PVP) and poly (ethylene glycol) diacrylate (PEGDA). We modified the silicone rubber using the prepared hydrogel coatings and cured it using a heating method. Then, we characterized its surface and evaluated the antifouling property, lubricious property, cytotoxicity, sensitization, and vaginal irritation. The results of water contact angle (WCA), protein adsorption, and friction coefficient indicated the success of the modification of the silicone rubber, leading to a significant decrease in the corresponding test values. Meanwhile, the results of cytotoxicity, sensitization, and vaginal irritation tests showed that the hydrogel coating-modified silicone rubbers have an excellent biocompatibility. This study describes how the silicone rubber could be modified with a biocompatible hydrogel coating. The hydrogel coating-modified silicone rubbers have improved antifouling and durable lubricious properties.

## 1. Introduction

Silicone rubbers are extensively utilized in medical applications due to their exceptional biological inertness, robust mechanical properties, chemical stability, and nonirritating, nontoxic nature towards human tissues [1,2,3,4]. These attributes make them suitable for both long-term implants and short-term indwelling devices within the body, such as urinary catheters, cardiac catheters, and defibrillators [5,6,7]. Despite these advantages, silicone rubber exhibits high frictional resistance, making insertion and removal difficult, which often leads to adverse symptoms, such as tissue damage and ulcers, in the medical process, imposing a significant strain on healthcare systems [8,9]. Therefore, it is essential to ensure that the outer surface of the silicone rubber catheter is adequately lubricated to minimize friction with surrounding tissues [10,11,12]. Furthermore, the silicone rubber catheters inserted into the body could easily absorb proteins, bacteria, and some biological cells, causing wound infection, inflammation, and thrombosis, due to their highly hydrophobic surface [13,14,15,16,17]. Hence, silicone rubber catheters with antifouling properties are urgently needed. 

Hydrogels have attracted considerable interest for use in clinical applications, such as wound healing [18,19,20,21], contact lenses [22,23,24,25], and tissue engineering [26,27,28,29], due to their hydrophilicity, softness, and biocompatibility, as well as maintaining the intrinsic properties of the substrate, such as its stiffness, strength, and toughness simultaneously [30,31]. Therefore, coating medical catheters with hydrogels has been recognized as an effective method to enhance the catheter’s surface properties and improve its overall functional performance. Ma et al. proposed a new method for preparing conformal hydrogel coatings on silicone rubber catheters [32]. The crucial process included shape-forming, gradient crosslinking by UV irradiation, and swell-peeling, resulting in a surface-bound, thin, and uniform hydrogel coating on silicone rubber catheters. Zhao et al. introduced a novel approach to securely attach tough hydrogels to elastomers by pretreating the elastomers with initiator benzophenone, which acted as a source of free radicals and a grafting agent, enabling a strong and stable bond between the hydrogels and the elastomer surfaces [33,34,35]. Li et al. utilized benzophenone solution and pre-gel solution followed by UV irradiation to obtain a PVAc primer layer. Then, the intermediate layer of poly (vinyl acetate) allowed the hydrogel to be grafted on the surface of rubber, forming a firm bond [31]. However, the above-mentioned process was tedious, and the residual benzophenone after UV light curing might have significant odor and yellowing problems. Hayeong Jang et al. constructed hydrophilic coatings based on PVP and PEGDA on polypropylene materials; the cured coatings exhibited superior adhesion than pure PVP coatings [36]. Nevertheless, their work did not investigate the lubricious property and durability of the coatings, and the network formed only by PEGDA through radical reaction could not firmly embed the PVP. Therefore, it is still a challenge to quickly and simply fabricate hydrogel coating-modified silicone rubber catheters with desired antifouling and lubricious properties.

**Scheme 1:**
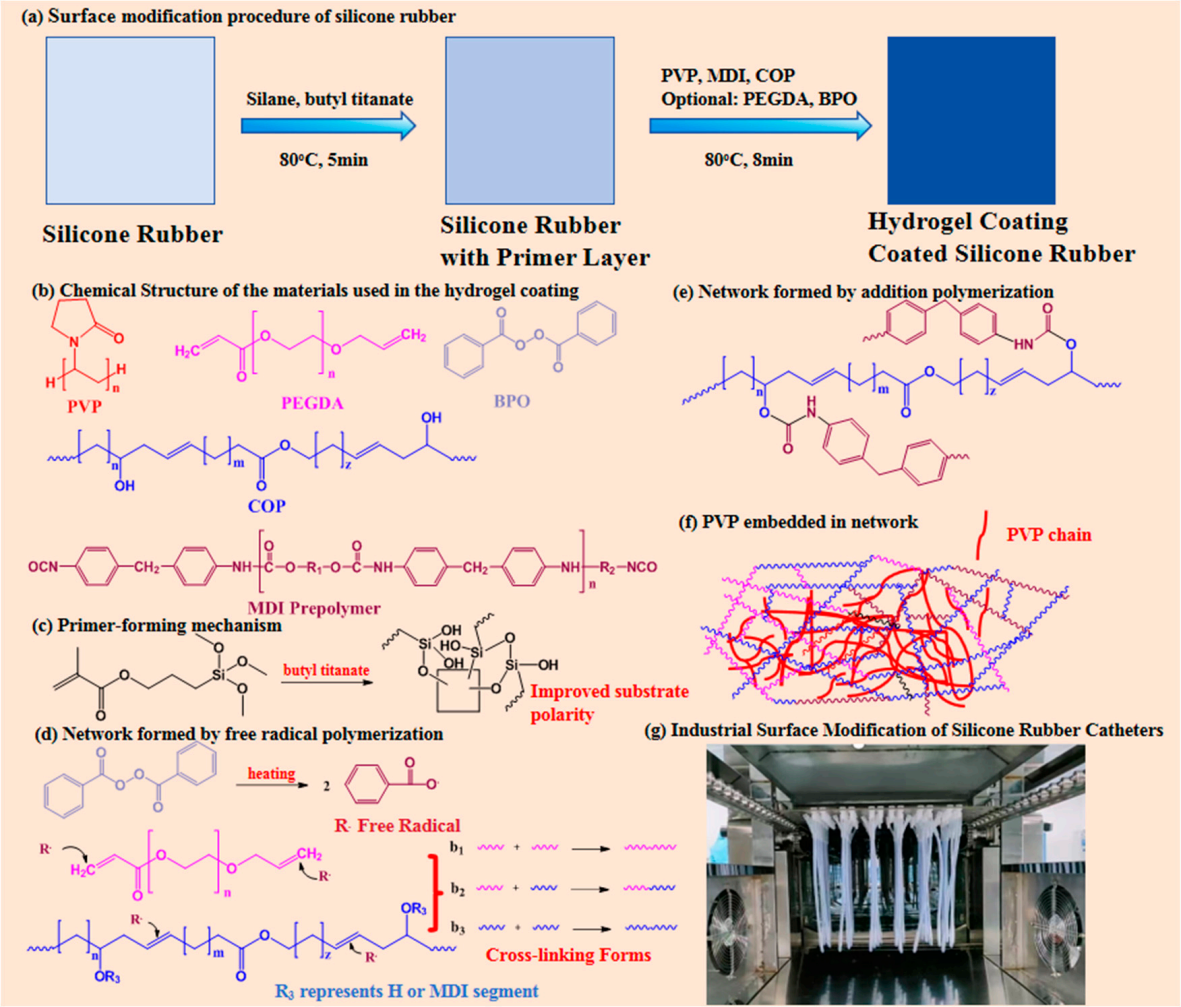
Schematic illustration of curing mechanism and application of the hydrogel coatings.

In this study, we presented a facile and effective silicone rubber surface functionalization strategy to develop a series of biocompatible hydrogel coatings with improved antifouling and durable lubricious properties on silicone rubber. The hydrogel coatings were based on PVP, diphenyl methane diisocyanate (MDI) prepolymer, castor oil polyol (COP), PEGDA, and dibenzoyl peroxide (BPO) and were denoted as PD-X (PD-0, PD-25, PD-50, and PD-100), in which X represents the amount of PEGDA relative to PVP in the coating. Under thermal curing conditions, cross-linked networks could be formed not only by MDI prepolymer and castor oil polyol’s addition reaction but also by castor oil polyol and PEGDA’s radical reaction under thermal initiation of dibenzoyl peroxide (Scheme 1). The surface of the silicone rubbers before and after hydrogel coating modification was evaluated by using a Fourier transform infrared spectrometer (FTIR), a scanning electron microscope (SEM), and a WCA test. Bovine serum albumin (BSA) was used to evaluate the nonspecific protein absorption of the modified silicone rubber sheet. The friction coefficient was tested to monitor the lubricious property of the silicone rubber catheter. The MTT assay method was further used to assess the cytotoxicity of the silicone rubber catheter. Finally, silicone rubber catheters modified with PD-50 hydrogel were prepared by industrial surface modification equipment, and the sensitization and vaginal irritation tests were estimated in an agency with China National Accreditation Service for Conformity Assessment (CNAS) certification. This work might provide a facile and efficient approach for mass production of antifouling and lubricious silicone rubber catheters. 

## 2. Results and Discussion

The silicone rubbers (sheets or catheters) were modified by a two-step dipping procedure followed by a thermal curing process. 3-Methacryloxypropyltrimethoxysilane, as a silane with moderate polarity, could undergo condensation reaction under the catalysis of butyl titanate to form a primer layer and introduce carbonyl groups and hydroxyl groups, which could improve the polarity of the silicone rubber. Therefore, the hydrogel coating could be evenly spread on the surface of silicone rubber, which was the prerequisite for effective bonding [37,38]. After thermal curing, the cross-linked network formed by thermal free radicals and thermal addition reactions could embed PVP in the coating and have effective bonding with silicone rubber. In the Results and Discussion section, silicone rubber without hydrogel coating modification was denoted as blank, and the modified silicone rubbers were denoted as PD-0, PD-25, PD-50, and PD-100 according to the corresponding hydrogel coatings.

### 2.1. Surface Characterization

#### 2.1.1. Fourier Transform Infrared Spectroscopy (FTIR) Test

**Figure 1:**
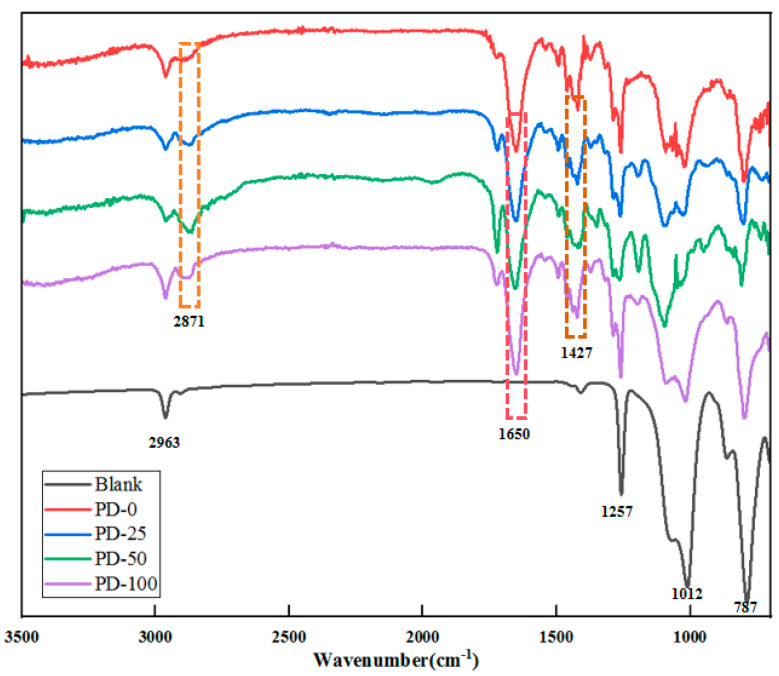
The infrared spectra images of blank and modified silicone rubbers.

The characterization of changes in the surface functional groups of hydrogel coating-modified silicone rubber was observed using FTIR. The infrared spectra images of the surfaces before and after hydrogel coating modification are shown in Figure 1. It could be observed that the characteristic absorption peaks of the blank silicone rubber included the Si-C stretching vibration peak (787 cm^−1^), Si-O stretching vibration peak (1101 cm^−1^), Si-CH3 absorption peak (1257 cm^−1^), and C-H asymmetric stretching vibration peak (2962 cm^−1^). After modification with hydrogel coating, the original characteristic absorption peaks of the blank silicone catheter were largely eliminated. The peaks observed of the hydrogel coating-modified silicone catheters were as follows: 1725 cm^−1^ for the polyurethane carbonyl stretching vibration peak, 1427 cm^−1^ for the N-H stretching vibration peak; 1650 cm^−1^ for the stretching vibration peak of the -CON- group of PVP; 1283 cm^−1^ for the C-N stretching vibration peak; and 2950 cm^−1^ for the C-H asymmetric stretching vibration peak. Meanwhile, with the increase in PEGDA in the hydrogel coating, the C-H symmetric vibration peak at about 2871 cm^−1^ was enhanced. Through infrared spectra testing, it is known that after coating with hydrogel, polyurethane film containing PVP and PEGDA moieties are formed on the surface of the blank silicone rubber, indicating a successful surface modification of the silicone rubber.

#### 2.1.2. Scanning Electron Microscope Test 

**Figure 2:**
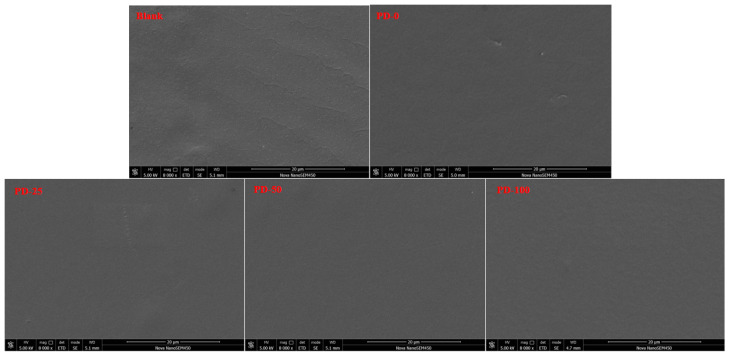
The SEM images of blank and modified silicone rubbers.

The surface morphology of the hydrogel coating-modified silicone rubber sheets was studied by SEM, using blank silicone rubber sheets as the control (shown in Figure 2). The blank silicone rubber sheet had a comparatively rough surface, while the hydrogel coating-modified silicone rubber sheets’ surface were smooth due to the adhered cured hydrogel coating.

#### 2.1.3. Water Contact Angles Measurement

**Figure 3:**
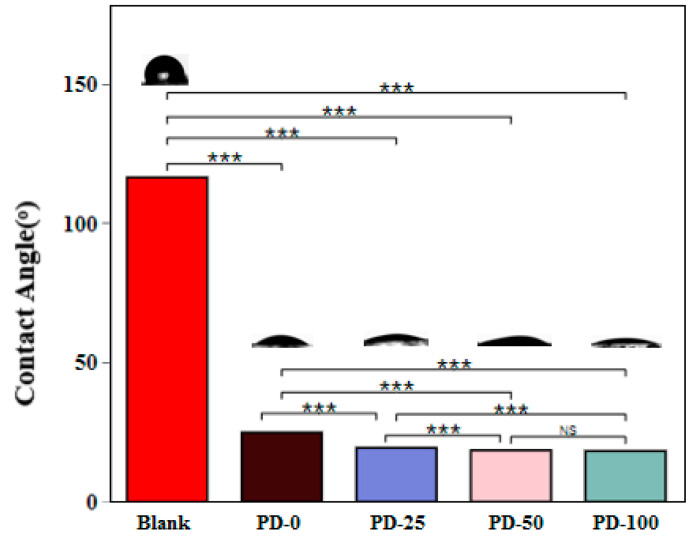
Water contact angle of blank silicone rubber and hydrogel coating-modified silicone rubbers. (*** *p* < 0.001, and NS means no significant difference).

The water contact angle is a crucial factor affecting the insertion characteristics of disposable medical devices, as it helps predict both the frictional forces and the potential tissue damage during insertion [39]. The hydrophilicity of the blank silicone rubber and hydrogel coating-modified silicone rubber sheets were characterized by WCA (Figure 3). The results showed that hydrogel coating-modified silicone rubber had significantly lower WCA than blank silicone rubber (highly hydrophobicity), which was attributed to the introduction of PVP and PEGDA, typical hydrophilic polymers. At the same time, as the amount of PEGDA increases, the contact angle further decreases due to the better hydrophilicity of PEGDA [36].

### 2.2. Protein Adsorption on Silicone Rubber

**Figure 4:**
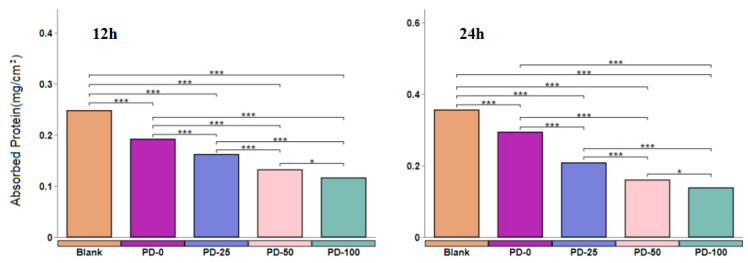
BSA adsorption on silicone rubbers after being treated with 5 mg/mL pure protein solutions for 12 and 24 h. (* *p* < 0.05, and *** *p* < 0.001).

Non-specific protein adsorption poses a significant challenge for the use of silicone rubber in vivo. To evaluate this issue on silicone rubber, bovine serum albumin (BSA) was used as a model protein. A BSA solution at 5 mg/mL in PBS was incubated with blank silicone rubber and hydrogel coating (PD-0, PD-25, PD-50, and PD-100) modified silicone rubbers for 12 and 24 h at 37 °C [40,41]. As illustrated in Figure 4, the PD-100 hydrogel coating exhibited the best antifouling property, which reduced the protein adsorption to 0.12 and 0.14 mg/cm² after 12 and 24 h, respectively. The PD-50 and PD-25 hydrogel coatings could also significantly reduce protein adsorption to 0.13–0.16 and 0.16–0.21 mg/cm^2^ under the same conditions. In contrast, the blank silicone surface adsorbed 0.25 and 0.36 mg/cm^2^ of BSA, while the surface of the PD-0 hydrogel coating was contaminated with adsorbed BSA at concentrations of 0.20 and 0.32 mg/cm², respectively. This indicated a notable reduction in protein adsorption with the hydrophilic hydrogel coatings, attributed to their increased surface hydrophilicity, as evidenced by a lower water contact angle. Among the hydrogel coatings, PD-100 was more effective at reducing protein adsorption compared to PD-0. After 12 h, the PD-0 hydrogel coating had 76% of the adsorption capacity of blank silicone, whereas the PD-100 hydrogel coating had only 46%. After 24 h, the PD-0 hydrogel coating maintained 82.6% adsorption of the blank silicone, while the PD-100 hydrogel coating further reduced to 38.8%. We attributed the enhanced antifouling properties of the PD-100 hydrogel to the improved surface hydration provided by PEGDA chain segments.

### 2.3. Lubricious Property Test

**Figure 5:**
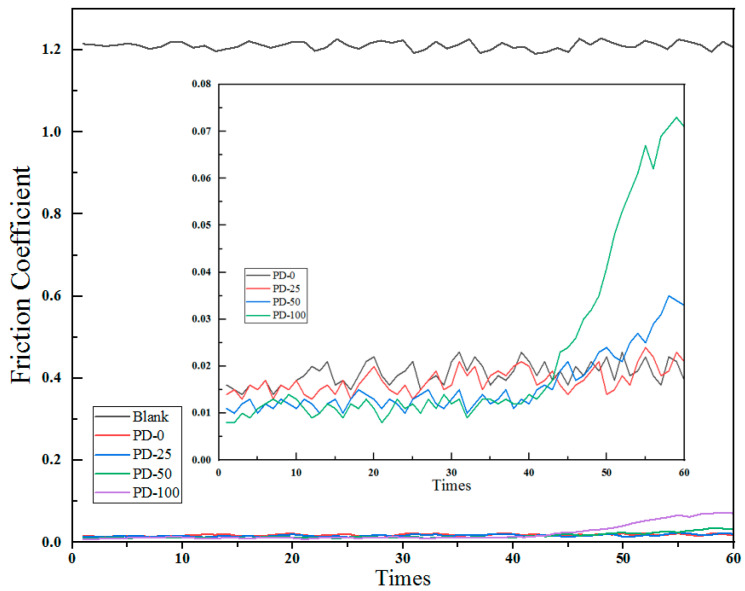
The friction coefficient test of blank silicone rubber catheter and hydrogel coating-modified silicone rubber catheters.

Lubricious property is a crucial issue for in vivo application of silicone rubber catheters. The hydrogel coating-modified silicone rubber catheter could significantly reduce tissue damage, thus enhancing patient safety and recovery times [42,43]. We tested the friction coefficient of blank silicone rubber catheters and hydrogel coating-modified catheters to evaluate this factor according to the ISO 8295-1995 standard [44]. As shown in Figure 5, it could be seen that the friction coefficient of hydrogel coating-modified silicone rubber catheters was significantly lower than that of blank silicone rubber catheters. After up to 60 tests, the friction coefficient of the catheters modified with PD-0 and PD-25 was still below 0.030, indicating good adhesion and durability of the coatings. Due to their better hydrophilicity, the PD-50 and PD-100 coating-modified catheters showed smaller friction coefficients in the initial and middle stages of the test. However, at the end of the test, the coefficient of friction of the catheters modified with both coatings increased, especially that of PD-100. This may be due to the increase in the PEGDA chain segments in the coating and its high hydrophilicity.

### 2.4. Cytotoxicity Assessment

**Figure 6:**
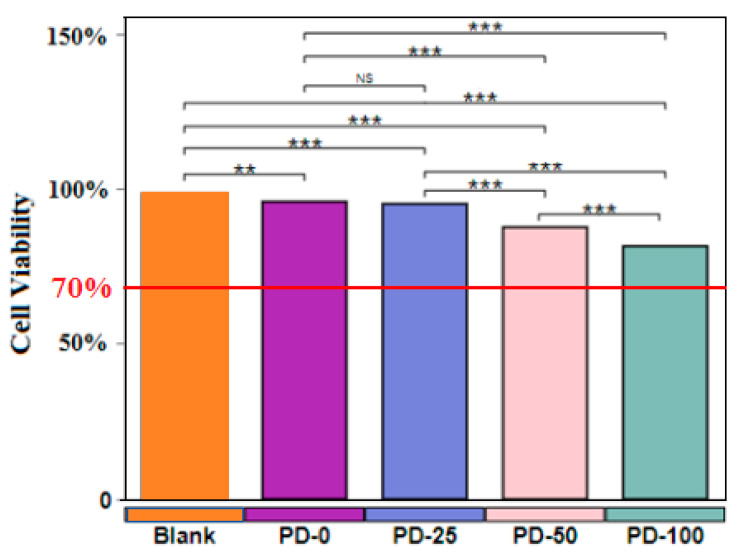
Cell viability of fibroblast L929 cells with blank silicone rubber and hydrogel coating-modified silicone rubber catheters. (** *p* < 0.01, and *** *p* < 0.001).

According to the ISO 10993-5:2009 (Biological evaluation of medical devices Part 5: Tests for in vitro cytotoxicity) [45], the cytotoxicity of the blank silicone rubber catheter and hydrogel coating-modified silicone rubber catheter extracts was examined using the MTT assay method, and L929 cells were used. As shown in Figure 6, it could be seen that the cell viability of the blank and hydrogel coating-modified silicone rubber catheters was above 70%, indicating that there was no obvious cytotoxicity to L929 cells. At the same time, it can be seen in Figure 6 that as the PEGDA content in the coating increased, the cell viability decreased, especially for PD-100. We assumed this result may be attributed to the coating falling off during extraction, which was consistent with the coating lubricious property test results, and the osmotic pressure of the extract increases, thus affecting the cell viability. 

### 2.5. Sensitization Test

**Figure 7:**
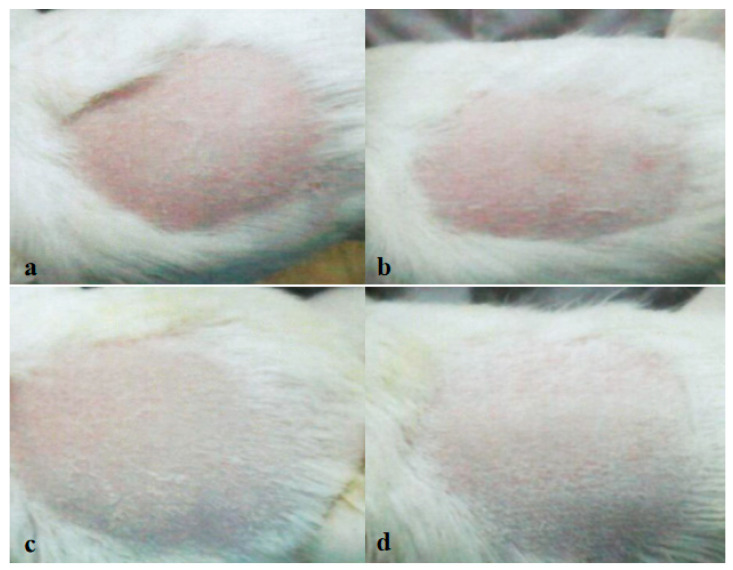
Clinical observation of different groups ((**a**): polar experiment; (**b**): polar control; (**c**): non-polar experiment; (**d**): non-polar control).

According to ISO 10993-10:2021 (Biological evaluation of medical devices Part 10: Tests for skin sensitization) [46], the potential of a PD-50 hydrogel coating-modified silicone rubber catheter to cause skin sensitization in guinea pigs was evaluated through the guinea pig maximum dose test. As shown in Table 1, the grading scores of the experiment and control group were 0 at both 24 and 48 h after stimulation according to the Magnusson and Kligman sensitization grading criteria, and there was no obvious abnormality in clinical observation (Figure 7). Therefore, the extract of the PD-50 hydrogel coating-modified silicone rubber catheter did not cause skin sensitization.

### 2.6. Vaginal Irritation Test

**Figure 8:**
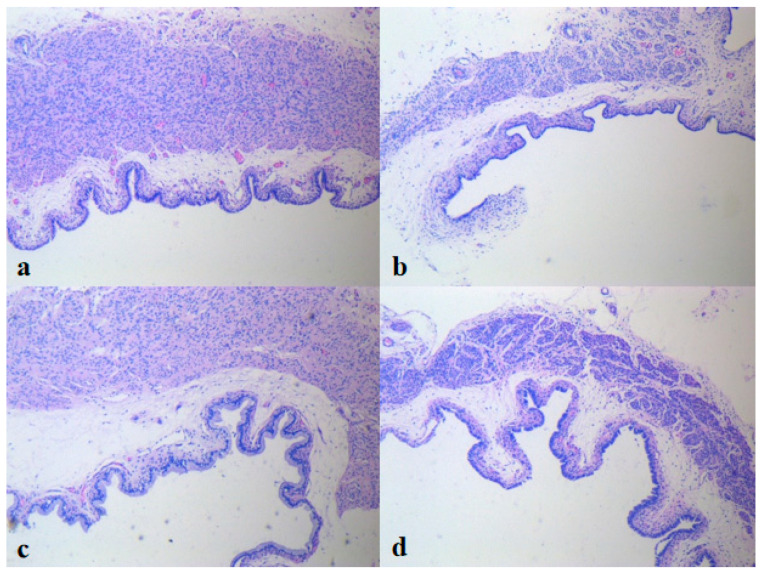
Pathological section pictures of different groups ((**a**): polar experiment; (**b**): polar control; (**c**): non-polar experiment; (**d**): non-polar control).

The purpose of this test is to evaluate the potential of the PD-50 hydrogel coating-modified silicone rubber catheter to produce vaginal tissue irritation under the test conditions. It could be seen from Table 2 that the average score of the experiment samples and control samples were 0; meanwhile, it could be seen that neither the experimental group nor the control group had inflammation from the pathological section pictures in Figure 8, which indicated the PD-50 hydrogel coating-modified silicone rubber catheter would not induce potential vaginal tissue irritation.

## 3. Conclusions

In summary, biocompatible hydrogel coating-modified silicone rubber was successfully fabricated with antifouling and lubricious properties. The FTIR, SEM, and WCA test results proved the successful modification of the silicone rubber. The hydrogel coating significantly improved the hydrophilicity of the silicone rubber and reduced the deposition of bovine serum albumin, which demonstrated improved antifouling property. Moreover, the friction test indicated that the silicone rubber modified with the hydrogel coating had reduced friction and good durability, which certified the network formed by addition reaction and free radical reaction could firmly embed the PVP. Finally, the results of cytotoxicity, sensitization, and irritation tests showed the hydrogel coating-modified silicone rubber catheter displayed excellent cytocompatibility, which had great potential for the development of medical catheters.

## 4. Materials and Methods

### 4.1. Materials

PVP(K90) was purchased from Boai Xin Kaiyuan Medical Technology Group Co., Ltd. (Jiaozuo, China); 3-Methacryloxypropyltrimethoxysilane (MPS) was purchased from Hubei Xinlantian New Material Co., Ltd. (Xiantao, China); MDI-based prepolymer was purchased from Huntsman Chemical Trading Co., Ltd. (Shanghai, China); Castor oil polyol was purchased from Itoh Oil Chemical Co., Ltd. (Yokkaichi, Japan); Heptane and butyl titanate were purchased from Chengdu Kolon Chemical Co., Ltd. (Chengdu, China); PEGDA, BPO and PDMS Sylgard^TM^ 184 Kit with the components of base and curing agent were purchased from Shanghai Aladdin Bio-Chem Technology Co., Ltd. (Shanghai, China); Silicone catheters were purchased from Haiyan Kangyuan Medical Instrument Co., Ltd. (Jiaxing, China). The hydrogel coating (PD-0) without PEGDA and BPO was prepared according to our previous literature [47].

### 4.2. Preparation of the Hydrogel Coating with PEGDA and BPO

PEGDA was added in various ratios relative to the concentration of PVP in PD-0. In our experiment, the PEGDA ratios were 25%, 50%, and 100 wt%, referred to as PD-25, PD-50, and PD-100, respectively. BPO was dissolved in 10 wt% anhydrous ethanol and then added to the PEGDA-containing hydrogel in an amount equal to 1 wt% of PEGDA. 

### 4.3. Preparation of the Silicone Rubber Sheet 

The PDMS SylgardTM 184 Kit was mixed in a 10:1 (*w*/*w*) base/curing agent ratio, stirred at 1000 rpm for 5 min, degassed for 6 min, and then the mixture was poured into the mold to a depth of 1 mm. The mixture was heated at 100 °C for 40 min. 

### 4.4. Preparation of Hydrogel Coating-Modified Silicone Rubber Catheter and Sheet

The silicone rubber catheters and sheets were dipped into a heptane solution of 2 wt% MPS and 0.25 wt% butyl titanate and then cured in an oven at 80 °C for 5 min so as to form a primer layer onto the silicone rubber. The treated silicone rubber catheters and sheets were dipped into the hydrogel coating, left to stand for 2 s, and then lifted out and placed in an 80 °C air drying oven to cure for 8 min. 

### 4.5. Surface Characterization of Silicone Rubber

The blank silicone rubber sheet and hydrogel coating-modified silicone rubber sheets were analyzed using the Nicolet iS10 Fourier transform infrared spectrometer (Thermo Scientific, Waltham, MA, USA). This procedure allowed for the characterization of changes in the surface functional groups of silicone rubber before and after modification.

The surface morphology of blank and hydrogel coating-modified silicone rubber were observed by the Nova NanoSEM 450 (FEI, Hillsboro, OR, USA). The surface wettability of silicone rubber was measured by the static WCA (Data Physics OCA, Filderstadt, Germany) using the sessile drop method with a 5 μL water droplet at room temperature. 

### 4.6. Protein Adsorption

The antifouling properties of blank silicones and hydrogel coating-modified silicones were assessed by a protein adsorption assay. Briefly, blank silicone rubber sheets and hydrogel coating-modified silicone rubber sheets were immersed in 3 mL of PBS that contained 5 mg/mL bovine serum albumin (BSA), and then all samples were incubated for 12 and 24 h at 37 °C. After incubation, the samples were rinsed several times with a PBS solution (pH 7.4) to remove unbound proteins. After cleaning, all blank silicone samples and hydrogel coating-modified silicone samples were immersed in 3 mL of a PBS solution containing 1.0 wt% sodium dodecyl sulfate (SDS), followed by ultrasonic treatment for 4 h to wash the adsorbed BSA. The SDS solution containing BSA was colored by adding the detection agent in the BCA protein assay kit. The absorbance of the solution at 562 nm was measured using Infinite F50 microplate reader (Tecan, Männedorf, Switzerland). Finally, the protein content of the solution could be calculated using the BSA standard curve.

### 4.7. Friction Coefficient Test 

Cut the silicone rubber catheter to a minimum effective length of 20 cm, and the friction coefficient of the catheter was measured using a friction coefficient testing instrument according to the ISO 8295-1995 standard. Take 2 test samples as a group, place the test catheter segment in the test fixture and fix them on the horizontal test bench, add an appropriate amount of 23 °C water on the horizontal test bench to at least cover the middle of the plane of the test sample tube body, and install and adjust the horizontal position of the slider (weight 200 g); start the friction coefficient testing instrument, let the slider slide a sufficient distance (preferably not less than 100 mm), and record the force relative to the displacement curve. After each test, clean the slider with clean water to avoid the influence of possible falling off of the coating on the test surface.

### 4.8. Cytotoxicity Test

According to the guidelines stated by ISO 10993-5:2009 to evaluate the cytotoxicity of the silicone rubber catheters. The test samples (blank and hydrogel coating-modified silicone catheters) were extracted in 10% FBS complete MEM medium for about 72 h in a 37 °C incubator with a ratio of 3 cm^2^/mL. The negative and positive control samples were extracted using the ratio of 3 cm^2^/mL and 6 cm^2^/mL, respectively at the same conditions. The medium control was incubated at the same conditions of extraction of the test article. L-929 cells (Wuhan Servicebio Biotechnology Co., Ltd., Wuhan, China) were seeded into 96-well plates and maintained in culture for about 24 h to form a nearly confluent monolayer, then exposed to test article extract. After 24 h exposure, the extracts were removed and the MTT solution was added to each well. The plate was incubated for 2 h. The MTT solution was then removed and replaced with 100 μL isopropanol. The absorbance at 570 nm was measured using Infinite F50 microplate reader (Tecan, Männedorf, Switzerland). The cell viability was calculated from the following Equation (1):

(1) $$Cell\;viability\left(\%\right)=\left[\frac{\left({OD}_{test}-{OD}_{nc}\right)}{\left({OD}_{pc}-{OD}_{nc}\right)}\right]\times 100\%$$

where OD_test_ is the absorbance of the liquid from the test group at 570 nm, OD_nc_ is the absorbance of the negative control group at 570 nm, and OD_pc_ is the absorbance of the positive control group at 570 nm. A cell viability of ≥70% is considered non-cytotoxic. 

### 4.9. Sensitization Test

PD-50 hydrogel coating-modified silicone rubber catheters were evaluated for the potential sensitization capacity in a guinea pig maximization sensitization test. The study was conducted in accordance with ISO 10993-10:2021. Under aseptic conditions, test catheters were sampled, and test parts were extracted with the concurrent extraction medium (saline and corn oil) at an extraction ratio of 3 cm^2^/mL and extracted at 50 °C for 72 h. A total of 40 guinea pigs, approximately 4–6 weeks old and weighing 335.20~481.11 g at initiation of dosing, were arbitrarily assigned to 4 groups (negative control: 7/group; test: 13/group). All animals were intradermally and topically inducted and then received a challenge patch. Each challenge site was scored at approximately 24 h and 48 h following challenge. The score is determined according to Table 3:

### 4.10. Vaginal Irritation Test

The test samples, PD-50 hydrogel coating-modified silicone catheters, were evaluated for the vaginal irritation in New Zealand white rabbits. The study was conducted according to ISO 10993-10:2021. The test samples’ extraction was injected into the rabbits’ vaginal to observe potential vaginal irritation reactions. The samples were tested using polar extraction medium, saline, and non-polar extraction medium, cottonseed oil. The extraction solution was gently inserted into the rabbit’s vagina using a syringe and connecting tubing, with 1 mL of sample extraction solution administered and maintained continuously for 5 days. The same method was applied to both control and test animals. 24 h after the initial contact and before each test operation, record the condition of the vaginal orifice and perineum. After the final contact, the animals were euthanized, and vaginal tissue was taken for pathological sectioning to observe the irritation condition. The control samples were handled in the same manner as the test samples.

Each tissue can be scored according to the scoring system specified in Table 4. The microscopic evaluation scores of the experimental group animals are added together and then divided by the total number of observations to obtain the average score of the experimental group. The maximum score is 16 (Table 5).

The experimental animals and procedures of the sensitization test and vaginal irritation test were approved by the Ethics Committee of the Tianjin Customs District Industrial Products Safety and Technical Center (permit number: 2300029). Throughout the experimental process, animal welfare concerns were well monitored.

### 4.11. Statistical Analysis

All data were expressed as mean ± standard deviation (SD) values. The T-test analysis was applied to confirm the statistical significance between groups; *p* < 0.05 was considered statistically significant (* *p* < 0.05, ** *p* < 0.01, and *** *p* < 0.001). In this study, at least three parallel experiments were executed for the assays.

## Figures and Tables

**Table 1:** Results of sensitization test.

Group	Number	Clinical Observation	Grading Scale	
			24 h	48 h
Polar experiment	1–10	No obvious abnormality	0	0
Polar control	11–15	No obvious abnormality	0	0
Non-polar experiment	16–25	No obvious abnormality	0	0
Non-polar control	25–30	No obvious abnormality	0	0

**Table 2:** Observation score of vaginal irritation test.

Group	Slice Position	Score				Average Score
		Epithelial	Leukocyte Infiltration	Vascular Congestion	Edema	
Polar experiment	Front/ Middle/ Back	0	0	0	0	0
Polar control	Front/ Middle/ Back	0	0	0	0	0
Non-polar experiment	Front/ Middle/ Back	0	0	0	0	0
Non-polar Control	Front/ Middle/ Back	0	0	0	0	0

**Table 3:** Magnusson and Kligman scale [45].

Patch Test Reaction	Grading Scale
No visible change	0
Discrete or patchy reythema	1
Moderate and confluent erythema	2
Intense erythema and/or swelling	3

**Table 4:** Tissue reaction microscopy scoring system.

Tissue	Reaction	Score
Epithelial	normal, intact	0
	cell degeneration or flattening	1
	tissue deformation	2
	local erosion	3
	extensive erosion	4
Leukocyte infiltration/ Vascular congestion/ Edema	none	0
	rare	1
	mild	2
	moderate	3
	severe	4

**Table 5:** Irritation reaction type.

Average Score	Reaction Type
0	none
5–8	mild
9–11	moderate
12–16	severe

## Data Availability

The original contributions presented in the study are included in the article, further inquiries can be directed to the corresponding authors.
